# Observations on Some Communications of Dr. Kinglake and Mr. Atkinson, Lately Published in the Journal

**Published:** 1816-11

**Authors:** 


*For the London Medical and Physical Journal.*

### *Observations on some Communications of Dr. Kinglake and Mr. Atkinson, lately published in the Journal;* by INTERROGATOR.

"Truth is, or ought to be, the object of every man's research."

THE controversy on midwifery practice, which has of late occupied so many of your valuable pages, appears not likely soon to terminate. Controversy is, perhaps, of all other things, the most likely means of eliciting truth. The human human mind, in its own passions and propensities, if undisturbed, becomes fixed and inveterate; and the hand of time has no other effect than to render prejudice more rooted; our own opinions are at least regarded as infallible; and, if the veil is attempted to be withdrawn, or even opposed to a stronger light, jealousy is awakened—becomes alarmed, and grasps with more tenacious firmness the cover by which it has unconsciously hidden its defects from public, and even from its own, inspection. But, by well conducted controversy, the mind feels the necessity of fixing its own positions, and is brought into opposition with other minds, whose ideas are jarring and discordant from its own; it is smoothed and softened by the concussion; error is convinced of its mistake; prejudice ridiculed into liberality; the steady, but unobtruding rays of truth emerge from the mass which had clouded its beauties, and receive the unconditional homage of men, who till then were unconscious of its beauties.

Both your controversialists (for I mean only to speak of Dr. Kinglake and Mr. Atkinson) seem to have run into extremes. The statement of Mr. A. on the authority of Mr. Hey, jun., gives a higher proportion of difficult cases than will be found to occur in general practice. The average of one in five, or one in seven, as far as my experience goes, is far too great. Dr. Bland's tables, quoted by Dr. Merriman, probably come pretty near mine. And as Dr. Kinglake seems decidedly of opinion that the fewer difficult cases occur in proportion as the attendant possesses less science, he must necessarily conclude this table to be rather in his favour than otherwise; the practice having occurred in the hands of midwives who would probably never think of the "newfangled eclat attached to the reputation of bad cases." It would be an unjust aspersion on Dr. K.'s humanity to suppose for an instant, if one case in eighteen, or in double or treble that number, were proved to require the assistance of the surgeon, and that without his aid, the life or future happiness of the patient would be hazarded; that he would place the practice in the hands of the present race of midwives for advantages merely visionary—to suppose, I say, that such a doctrine should hold a place in his mind, would be an imputation equally discreditable to him as a man and as a practitioner.

The undisputed fact of parturition taking place with so much ease amongst females living according to nature, and upon which the doctor has rung so many and such varied chimes, is an argument scarcely applicable to the subject. That they require no aid from the accoucheur, we are not disposed to dispute, nor are we disposed to claim the privilege of troubling them with our assistance. But, to the fair females who so eminently adorn and beautify society in these islands, we shall continue to give that help which the hand of science only, in many instances, is capable of affording.

Dr. K. will find his cause (if a good one, which I have no doubt he believes it to be) much more effectually advocated by a clear and simple statement of facts, than by his luxuriant periods about Nature's all-sufficiency, and "Nature's simple, comprehensive, and efficient operations." If he would only furnish us with the number of cases which his thirty, forty, and fifty years old friends have had subjected to their observation, we might, perhaps, feel inclined to join in the doctor's offerings at Nature's shrine.

No one will dispute Dr. K.'s position, that "human nature is universally the same; certain local modifications and influences may vary the external aspect of circumstances, but vitally and essentially the same fundamental principles exist." If this means, human nature is the same, but by certain local modifications and influences she is rendered not the same, it must be agreed to, nem. dis. But, sir, permit me to caution you, lest the violence of debate from the different conclusions formed upon our heads, should fire some of our gunpowder spirits and set them a *mangling* the outside of each other's *noddles*. If the doctor but knew the especial care and regard which we bestow on this important part of our bodies, and as he professes such a horrid antipathy to boring fœtal skulls, I doubt not he would have mercy on the more important heads of adults. The benefit too would be mutual, as easing the doctor as well as ourselves. His mercy would be truly

"Twice blessed,
"It blesseth him that gives, and him that takes."

I must now beg pardon for obtruding myself again so soon upon your notice, and encroaching so far upon your pages. I have gone further than I intended, but hope you will deem the importance of the subject a sufficient apology.

*Rochdale; October* 6, 1816.

We are much obliged to our humorous correspondent for these effusions of wit, and still more for reminding us of the danger lest a most interesting controversy should become diffuse and unconnected; most of all lest it should degenerate into personality. While, therefore, we return sincere thanks to the numerous correspondents who have honoured us with their communications, we are obliged to advertise them that we can no longer spare room for any thing but records or facts, well-authenticated, and with the signature of the parties.—Edit.

